# Editorial: Nanomedicine development and clinical translation

**DOI:** 10.3389/fchem.2024.1458690

**Published:** 2024-10-08

**Authors:** Chuanqi Peng

**Affiliations:** SalioGen Therapeutics, Lexington, MA, United States

**Keywords:** nanomedicine, nanoparticle, drug development, therapy, clinical translation

## Introduction

Nanomedicine, under the spotlight of biomedical research, has embraced rapid evolution and considerable accomplishments in the fundamental discovery and drug development for potential clinical translation. For the past decades, many novel concepts have been proposed by using engineered nanoparticles or nanomaterials for drug delivery, disease prevention, intervention and monitoring. Given the unique physicochemical properties, nanoparticles protect the payload (therapeutic or imaging agents, nucleic acids, etc.) from degradation, fine-tune the *in vivo* transport for targeting and clearance, modulate the controllable payload release (Gawne et al., 2023). From the very first FDA-approved nano-drug in 1995 – Doxil (in the form of PEGylated liposomal doxorubicin) (Barenholz, 2012) to now broadly applied lipid nanoparticle-based COVID-19 mRNA vaccines (under emergency use authorization in 2020) (Polack et al., 2020; Baden et al., 2021), nanomedicines have not only improved the life quality of patients bearing cancers, rare genetic disorders (Adams et al., 2018) and other diseases, but also protected healthy people effectively from viral infection and resulting complications. Over a hundred other candidates are being investigated in clinical trials as nanoparticle vaccines, therapies and diagnostics (Anselmo and Mitragotri, 2021).

Meanwhile, the overall success rate falls short in the clinical translation of nanomedicines. And the clinically approved nanomedicines are mostly in the context of liposomes or lipid-based nanocarriers. In many research studies, the strategies in nanomedicine design, material synthesis and analytics often lack the consideration of translation potential or clinically relevant solutions (Liz-Marzán et al., 2022). Notably, the practice of transforming an interesting concept into a scalable, applicable and marketable medical product is quite challenging, as its development may be hampered or terminated at any time due to the limitations towards preclinical proof of concept, chemistry, manufacturing and controls (CMC), clinical safety and efficacy, regulatory compliance and even post-approval. So, nanomedicine researchers have recently concluded a series of developmental barriers to overcome (Anchordoquy et al., 2024), including the formulation, toxicological, physiological, immunological, and translational barriers. More importantly, the successful clinical translation involves extensive collaborations with pharmaceutical, clinical and regulatory groups in the assessment of drug’s critical quality attributes, pharmacokinetics, dosimetry, therapeutic index, cost-effectiveness as well as consideration of the complexity of a disease.

## Highlights of contributions to the Research Topic

We opened the Research Topic on *Nanomedicine Development and Clinical Translation* last year, aiming to better bridge the basic science research and clinical translation for current nanomedicine development. Herein, our hope is to receive the input from innovative researchers pinpointing the underlying challenges and opportunities of nanomedicine, so that clinical translation of these nano-products may be accelerated and become successful more broadly. As a result, we have received and published four peer-review articles (in original research and review papers) in this Research Topic collection.

One intriguing feature of nanoparticles is that once administered, they accumulate preferentially in tumor environment through leaky tumor vasculature or transcytosis. When it comes to the hard-to-target tumors like gliomas, nanoparticle-based drug delivery systems (NDDS) may still present enhanced tumor targeting compared to most chemotherapeutic drugs. The review article by Lai et al., put forward the pathophysiology of gliomas involving the blood-tumor barrier, discussed the nanoparticle-based delivery mechanisms to gliomas, and then overviewed various types of NDDSs for glioma treatment and their preclinical progress. The authors also provided a summary of nanoparticle-based drug carriers investigated in clinical trials, their challenges in clinical translation and prospectives in future development.

A second piece of paper shared the thoughts on the application of nanotechnology to the prevention, diagnosis and management of orthopedic disorders. The review by Liang et al. covered how nanostructured biomaterials can be applied to orthopedic conditions, such as prosthetic replacement of joints and treatment of osseous and chondral defects. The authors further reviewed the nanomaterials for bone restoration and osteoporosis prevention, orthopedic surgery sensor and implantation. Some emerging nanocomposites may offer enhanced mechanical properties and biocompatibility, meanwhile, the assessment of toxicity and cost should also be considered.

The next contribution to this Research Topic reported the green synthesis and *in vitro* study of a drug-loaded nanocomposite for photodynamic therapy. In the research article by Karimi et al., the DOX-loaded MgO/C-dots nanocomposite was prepared and characterized, and its *in vitro* photodynamic activity (in addition to chemotherapy) was investigated against C26 colorectal cancer cells with illumination of UV or red light. The fluorescence resonance energy transfer (FRET) from MgO NPs to DOX as well as the persistent afterglow for up to 200 s were observed. The photocatalysis and *in vitro* cell-based experiments suggest the possibility of carrying out photodynamic therapy with the nanocomposite for superficial cancers, and more related studies may be expected in future.

We also include another interesting research article by Mondal et al. In the study, the authors dived deep into the structure-function relationship in gold nanoparticle (AuNP) synthesis. They developed a new linker strategy for AuNP surface modification by replacing the chiral lipoic acid (LA) with achiral isolipoic acid (iso-LA), in order to improve nanoparticle stability, resolve the “contamination” from unnatural LA isomer and prevent unwanted immune responses *in vivo*. The authors synthesized Thomsen-Friedenreich antigen disaccharide (TF_ag_) bearing AuNPs with serine and threonine glycoamino acids as linkers, which showed both good stability *in vitro* and biological activity in binding to Gal-3. These nanoparticles may be applied to the design of antimetastatic therapeutics that could benefit from linear and achiral geometrical structures.

## Summary

Nanomedicine is built into better understanding nano-bio interactions and offering profound solutions to diseases and patients. We hope this Research Topic serves as a good practice in continuing the push for basic research in nanomedicine, and also brings up increasing attentions and thoughts on the clinical relevance. The joint efforts in nanoscience, manufacturing, pharmaceutical and medical development would overcome the multiple obstacles in the critical path of nanomedicine translation.
